# Effectiveness of an Adapted Physical Activity Protocol for Upper Extremity Recovery and Quality of Life Improvement in a Case of Seroma after Breast Cancer Treatment

**DOI:** 10.3390/ijerph17217727

**Published:** 2020-10-22

**Authors:** Daniela Mirandola, Francesca Maestrini, Giuditta Carretti, Mirko Manetti, Mirca Marini

**Affiliations:** 1 Department of Experimental and Clinical Medicine, Section of Anatomy and Histology, University of Florence, 50134 Florence, Italy; daniela.mirandola@unifi.it (D.M.); giuditta.carretti@gmail.com (G.C.); mirko.manetti@unifi.it (M.M.); 2 The Italian League against Tumors (LILT), 50126 Florence, Italy; francescamaestrini19@gmail.com

**Keywords:** adapted physical activity, breast cancer, seroma, upper limb disability, cancer survivorship, quality of life

## Abstract

Growing evidence indicates that physical activity (PA) interventions may reduce upper limb function-limiting side effects of treatments and improve quality of life (QoL) of breast cancer (BC) survivors. However, the possible effectiveness of PA in cases developing seroma after BC treatment has yet to be demonstrated. Here, we describe for the first time the impact of a structured PA pathway (i.e., two cycles of eight-week adapted PA followed by eight-week adapted fitness) on upper limb disability and QoL in a peculiar case of chronic seroma as complication of reconstructive plastic surgery after left breast mastectomy and lymphadenectomy. A 56-year-old female BC survivor underwent a functional test battery (i.e., shoulder–arm mobility, range of motion, back flexibility and indirect assessment of pectoralis minor muscle) at baseline, during and after ending the structured PA pathway. Upper limb and back pain intensity and QoL were evaluated by numerical rating scale and Short Form-12 questionnaire, respectively. A relevant seroma reduction, an improvement in upper limb mobility and pain perception, and an overall increase in QoL were achieved after the structured PA intervention. Our findings suggest that an adapted PA intervention may represent an effective strategy for seroma treatment in BC survivors.

## 1. Introduction

Breast cancer (BC) is the most frequently diagnosed neoplasm among women today and although the incidence has increased over the past decade, the mortality has gradually declined. Indeed, survival increase can be attributed to earlier diagnosis as well as improved therapies [1,2,3,4]. Surgical intervention is currently the main effective treatment for BC and can be complemented with radiotherapy, chemotherapy, hormone therapy and/or biological therapy. Despite substantial advances in technology, the frequent association of surgical procedures with other treatments, and the use of more personalized/less extensive surgical approaches, BC surgery-related complications are still observed [3,5]. The different surgical interventions include simple mastectomy or combined with reconstruction that may influence the patient’s motor function soon after the treatment or years later [6]. Extensive surgical approaches including axillary lymphadenectomy cause more severe upper limb dysfunctions such as lymphedema, reduced shoulder range of motion (ROM) and strength, pain, and limitations in performing activities of daily living [4]. Even if breast reconstructive surgery combines together esthetic and psychological benefits, per se it can also induce possible functional sequelae that, added to those related to BC surgery, negatively impact patient’s quality of life (QoL) [6]. In a long-term perspective, such physical side effects frequently generate chronic pain, low back pain and reduced trunk flexion [6]. In this context, the pathophysiology of breast seroma is being increasingly discussed in the literature. In particular, studies on mastectomy implicate that lymphatic disruption, ongoing inflammation, foreign bodies, and axilla movement may cause persistent exudate and fluid accumulation in dead spaces, thus causing seroma. Furthermore, another seroma developing area is the peri-prosthetic one [7]. Even if seroma is a common complication during the immediate postoperative period, it is extremely rare as a late complication of breast implant [8,9]. Moreover, clinical findings have shown that a seroma can develop months and years later than surgery [7,8]. Although many seroma patients are asymptomatic, some of them experience persistent pain, shoulder dysfunction, paraesthesia and need persistent fluid aspiration for months. Traumatic aspiration may increase the incidence of surgical site infection, clinic visit, and mental stress of patients [8]. While recent research supports that physical activity (PA) interventions can improve physical functioning, treatment-related symptoms, and QoL in cancer survivors [3,5,10,11], the possible effectiveness of PA in cases developing seroma has yet to be demonstrated [12]. Therefore, we herein evaluated for the first time the possible effects of a structured PA pathway for upper extremity recovery and QoL improvement in a case of peri-prosthetic seroma after BC treatment.

## 2. Materials and Methods

### 2.1. Case Description

A 56-year-old married woman was referred to the Cancer Rehabilitation Center (Ce.Ri.On) in Florence for BC-related follow-up management. In particular, the patient had an increased breast swelling for several years causing tension and pain especially on the left side of the thorax. The survivor was included in the Ce.Ri.On waiting list for a possible rehabilitation program, and then randomly recruited to initiate an adapted PA (APA) intervention. In September 2018, on the basis that there was no medical contraindication, the Ce.Ri.On rehabilitation physician recommended the woman’s participation in a well-planned and structured PA pathway consisting of two cycles of eight-week APA followed by eight-week adapted fitness (AF) in order to reduce breast swelling and pain, and to improve shoulder movement. Tracing the clinical history, in November 2014 mammogram findings showed a suspicious lump on the upper quadrant of the left breast in the asymptomatic patient without family history of cancer. Ultrasound-guided core biopsy revealed an invasive mammary carcinoma. Next month, nipple-sparing mastectomy and axillary lymph node dissection was performed, and immediate tissue expander (i.e., Allergan 300 SV) reconstruction was also executed. The histopathologic report was an infiltrating ductal carcinoma grade 3. Subsequently, owing to early nipple necrosis postoperatively, 10 sessions of hyperbaric oxygen therapy were administered. Moreover, the patient received BC adjuvant treatment such as four chemotherapy cycles, two radiotherapy sessions and hormone therapy (i.e., letrozole) for about five years. In December 2015, capsulectomy with tissue expander removal and prosthesis (i.e., Allergan 280 MM) insertion were performed. Concurrently, contralateral prophylactic mastectomy for calcifications followed by immediate reconstruction with permanent prosthesis was also carried out. In September 2016, a postoperative persistent seroma developed, requiring percutaneous drainage every two/three weeks. The diagnosis of seroma formation was also confirmed by magnetic resonance imaging. In March 2017, in order to solve this clinical sequela, further bilateral capsulectomy, implant removal and replacement by more voluminous prosthesis (i.e., Allergan 375 MF) were performed. However, a month later, seroma reappeared in the left side. In fact, copious amounts of fluid (i.e., 180–200 mL) were frequently aspirated. Cytology and histology of accumulated fluid showed negative results. In August 2017, the seroma localized to the left side had not improved. In particular, the patient was physically active and referred a worsening of symptoms after swimming. The seroma was aspirated once again, and the cytological examination of the fluid showed no sign of bacterial growth. Then, from September 2017 to February 2018, lymphatic drainage was prescribed three days per week. Afterwards it was suspended because the ultrasound confirmed the persistence of seroma between the implant and the capsule (thickness 1 cm). Noteworthy, all treatments were suspended because the seroma, although not solved, remained stable in absence of minimal effort. Finally, the patient was referred to an adapted exercise specialist (D.M.) in September 2018 and executed a structured PA pathway. Physical assessment and two cycles of eight-week APA program were carried out at the Ce.Ri.On center between October 2018 and January 2019. After APA ending, an AF protocol was properly continued outside the Ce.Ri.On from February 2019 to March 2019. To date, in order to maintain the benefits achieved by following a structured PA pathway, the subject continues the AF activity. At baseline, during and after ending the structured PA intervention, the woman underwent a functional test battery to assess the upper limb mobility through active ROM test and muscle length test [3,5,13]. Active ROM test was executed with the subject standing by goniometry taking into account the extension (range 0–45°), flexion (range 0–180°), external rotation (range 0–90°), and abduction (range 0–180°) [3,13]. Muscle length test was performed with the subject in a supine position by elevating the arm and measuring the distance (cm) from the lateral epicondyle to the surface, with a smaller distance from the surface corresponding to a greater upper limb mobility [13]. Moreover, for assessing lumbar and hamstring ROM, a sit and reach test was used [3,5,13]. To perform this test, the subject sits with legs extended as straight as possible and then flexes the hip joints and vertebral column trying to touch the toes in dorsiflexion with the fingertips of both hands simultaneously. A meter rule is placed between the legs with 0 cm located at the heel line to measure the distance from toes to hands [13]. Indirect assessment of pectoralis minor muscle (PMm) length test was also applied by setting the subject in supine position and measuring the distance between treatment table and acromion. In particular, a forward shoulder/scapular posture is frequently related to a tight PMm [14]. In addition, the subject filled in the Short Form-12 (SF-12) questionnaire and the numerical rating scale (NRS) to evaluate health-related QoL and to assess back and shoulder pain intensity, respectively [3,5,13]. The SF-12 questionnaire consists of a physical component score and a mental component score. For both components, higher scores indicate a greater QoL [13]. The NRS evaluates pain intensity on a 0–10 scale (0 = no pain, 10 = worst imaginable pain) [13]. A signed informed consent form was obtained from the participant in accordance with the Declaration of Helsinki.

### 2.2. Structured Physical Activity Protocol

**Figure 1:**
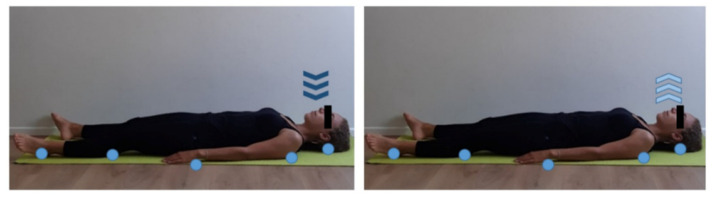
Body perception/awareness exercise on the floor. Supine position, rest straight arms by body sides and hand palms on the floor, eyes closed. Keep a natural pace of inhalation (dark blue arrows) and exhalation (light blue arrows). Focus on body’s contact points on the floor (light blue dots) and analyze differences between right and left hemisoma. Five min work at the beginning of training session. Repeat at the end of training session and compare body perception.

The structured PA protocol was planned by an adapted exercise specialist (D.M.) and was tailored to the specific patient’s needs on the basis of baseline assessment results. In particular, an increase in upper limb mobility, a reduction in pain perception, and an overall improvement of QoL were set as specific objectives to achieve. This protocol was organized in three cycles of eight-week training each, namely two cycles of APA (i.e., APA_1_ and APA_2_) followed by one cycle of AF, and consisted of one-hour sessions scheduled during two nonconsecutive days per week. In the first part of APA_1_ (from the 1st to 6th session), in order to improve the subject’s body conscious awareness, supine position exercises (Figure 1), as well as additional exercises in different positions (i.e., orthostasis and sitting), were proposed.

**Figure 2:**
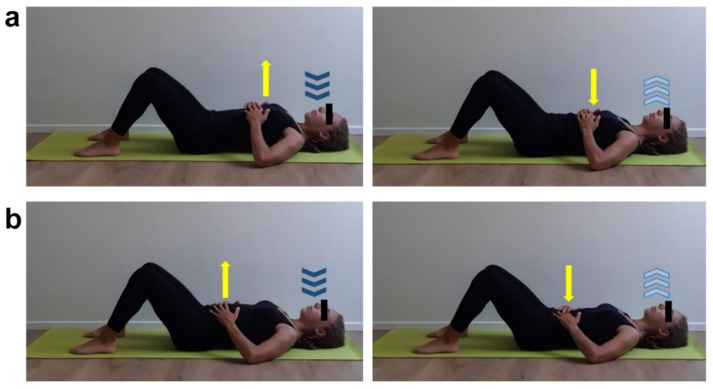
Breathing exercises. (**a**) Supine position, flexed legs. Focus on costal breathing by placing open hands on last ribs and perceiving chest expansion/retraction (yellow arrows). Inhale (dark blue arrows) through the nose, direct the breath to the center of chest and exhale (light blue arrows) through the mouth. Five min work. (**b**) Supine position, flexed legs. Focus on diaphragmatic breathing by placing open hands on abdomen. Inhale (dark blue arrows) through the nose and perceive abdomen lifting upward (yellow arrow), direct the breath to lower chest area and exhale (light blue arrows) through the mouth perceiving abdomen getting downward (yellow arrow). Five min work.

Subsequently, a development/improvement of breathing perception and control was aimed through intercostal and diaphragmatic breathing exercises (Figure 2).

**Figure 3:**
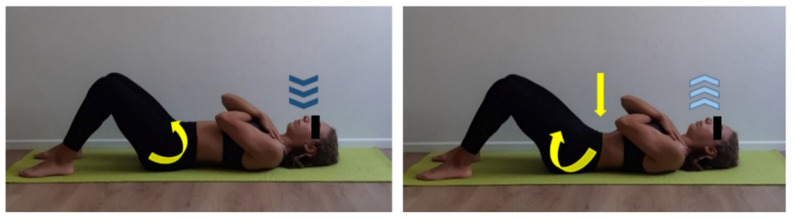
Lumbar mobilization exercise. Supine position, flexed legs. Cross both upper limb on chest and place each hand on contralateral shoulder. Inhale (dark blue arrows) through the nose while performing a pelvis anteversion (curved yellow arrow) and exhale (light blue arrows) through the mouth while performing a pelvis retroversion (curved yellow arrow). Focus on perceiving abdomen getting downward during exhalation phase (yellow arrow). Five min work.

**Figure 4:**
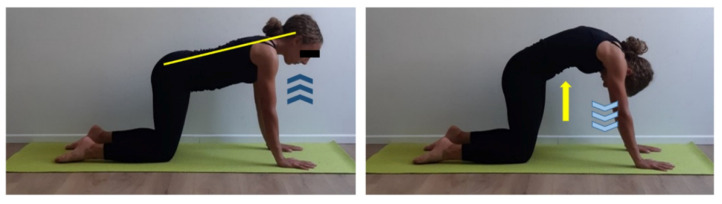
Spine mobilization exercise in quadruped position. Quadruped position, neutral spine attitude (yellow line), set hands shoulder-width apart and knees and feet hip-width apart. Inhale (dark blue arrows) then exhale (light blue arrows) and perform a posterior pelvic tilt and a head flexion (yellow arrow), inhale, and get back to start position paying attention not performing an anterior pelvic tilt. Two sets of 10 repetitions, 30 s recovery.

Tactile and verbal cues were incorporated to encourage diaphragmatic excursion during inspiration and promote a relaxed expiration. Indeed, a good coordination between the performed movements and inhalation/exhalation phases increases the exercise effectiveness. Spine and pelvis mobilization exercises were also performed (Figure 3 and Figure 4).

**Figure 5:**
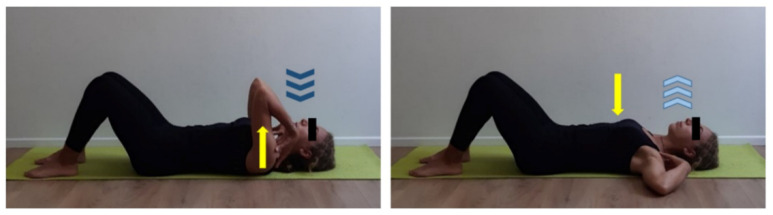
Scapular mobilization exercise in supine position. Supine position, flex both legs, set feet hip-width apart. Place both hands fingertips on shoulders by rising upper limbs on sagittal plan (yellow arrow) and flexing forearms. Inhale (dark blue arrows) then exhale (light blue arrows) and perform a scapula adduction by opening flexed upper limbs floorward keeping fingertips in contact with shoulders. Get back to start position. During exhalation phase focus on keeping the spine in touch with the floor without lifting thorax (yellow arrow). Three sets of 10 repetitions, 30 s recovery.

**Figure 6:**
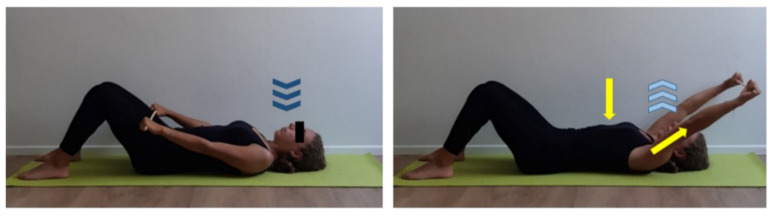
Shoulders mobilization exercise paired to breathing. Supine position, flexed legs, set straight arms by body sides with relaxed shoulders, holding a stick without forcing the grip. Inhale (dark blue arrows), exhale (light blue arrows) and simultaneously flex straight arms upward (yellow arrow) and get back to start position. During exhalation phase focus on keeping the spine in touch with the floor without lifting thorax (yellow arrow). Two sets of 10 repetitions, 30 s recovery.

**Figure 7:**
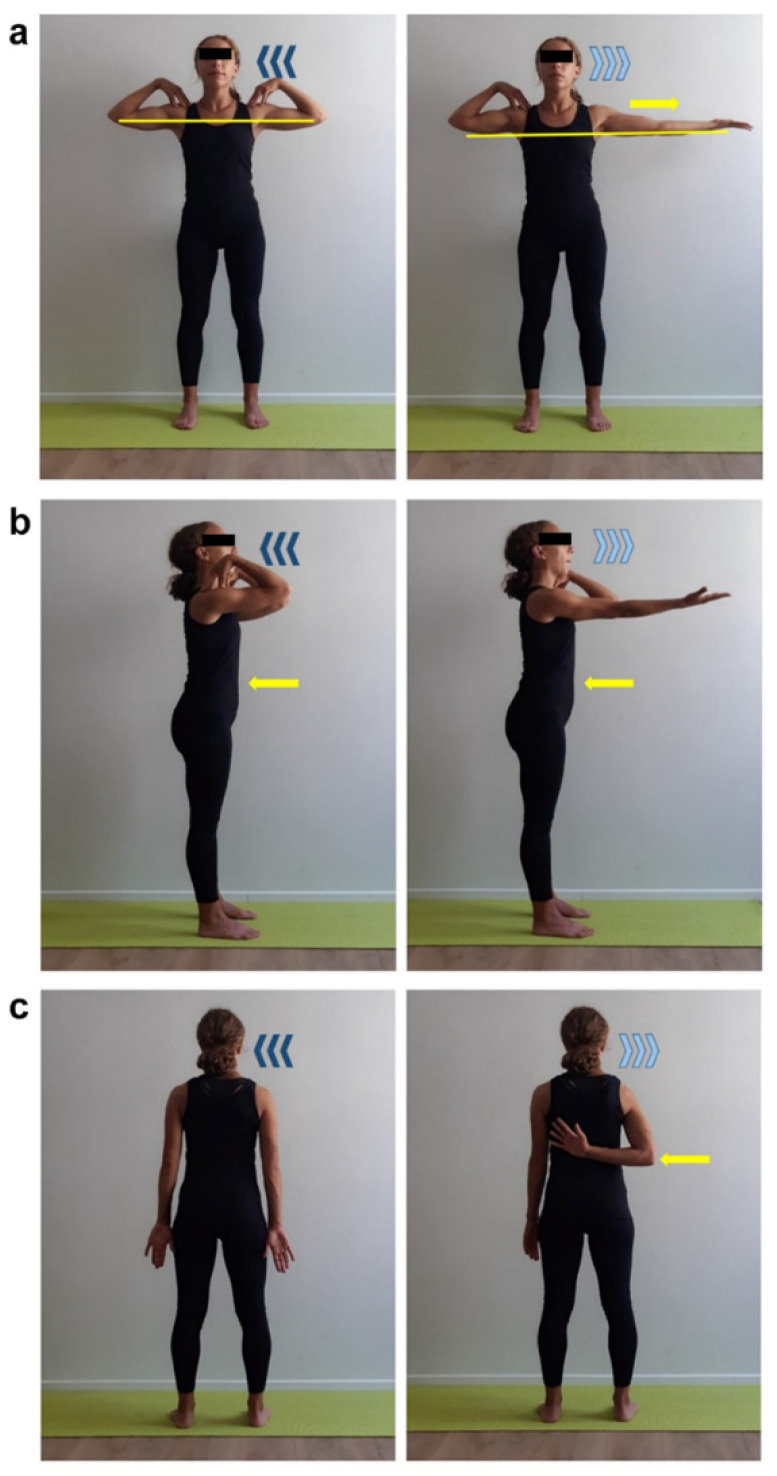
Upper limbs mobilization exercises on different plans. (**a**) Orthostatic position, lift straight arms out at body sides until reaching shoulders level (yellow line), keep gaze and upper limbs parallel to the floor and flex forearms. Touch both shoulders with ipsilateral hand’s fingers, activate core muscles and inhale (dark blue arrows). Keeping this position, exhale (light blue arrows) and alternate right and left forearm extension/flexion on arm. Focus on forearm extension performing a complete movement from elbow to fingertips (yellow line and arrow). Three sets of 30 s repetitions, 15 s recovery. (**b**) Orthostatic position, repeat previous exercise on sagittal plan, focusing on core muscles activation (yellow arrow). Three sets of 30 s repetitions, 15 sec recovery. (**c**) Orthostatic position, set straight arms by your sides with pronated hands. Inhale (dark blue arrows), exhale (light blue arrows), bend one forearm at a time behind back, trying to touch the inferior angle of opposite scapula with the back of the hand. In order to achieve a higher range of motion, keep elbow close to the trunk (yellow arrow) first and then flex forearm. Three sets of 30 s repetitions, 15 s recovery.

In the last part of APA_1_ (from 7th to 16th session), upper limb mobility exercises were proposed, setting a special focus on scapulohumeral joint’s ROM and PMm’s functionality and activation improvement (Figure 5, Figure 6 and Figure 7).

**Figure 8:**
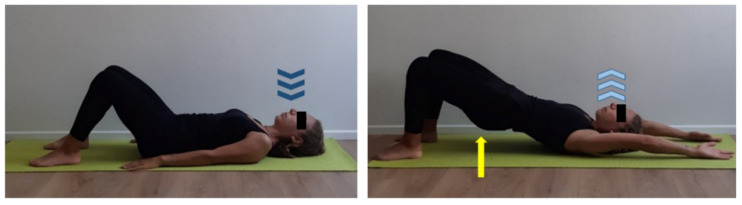
Glutes strengthening exercise paired to shoulders mobilization. Supine position, flex both legs, set feet hip-width apart close to glutes, rest straight arms by body sides and hand palms on the floor. Inhale (dark blue arrows), exhale (light blue arrows), lift glutes (yellow arrow) performing a bridge and simultaneously flex straight arms upward until reaching the floor with the back of both hands. Inhale and get back to start position activating abdominal muscles contraction. Two sets of 10 repetitions, 1 min recovery.

**Figure 9:**
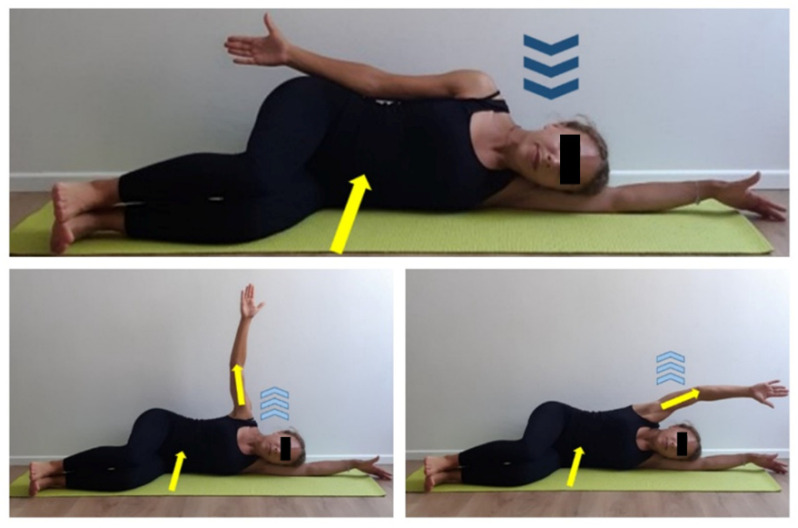
Scapulohumeral mobilization exercise in lateral decubitus position. Lateral decubitus, both legs and thighs 90 degrees flexed. Keep floor arm straight upward and rest head on it. Set top arm straight on body side, hand palms facing forward. Activate core muscles (yellow arrow). Inhale (dark blue arrows), exhale (light blue arrows) and perform a top upper limb abduction (yellow arrow) until bringing it parallel to the floor one (yellow arrow). Imagine hand is drawing a big semicircle during arm movement and focus on keeping upper limbs stretched and core muscles activated (yellow arrows). Inhale and slowly get back to start position. Two sets of 8 repetitions, 30 s recovery.

**Figure 10:**
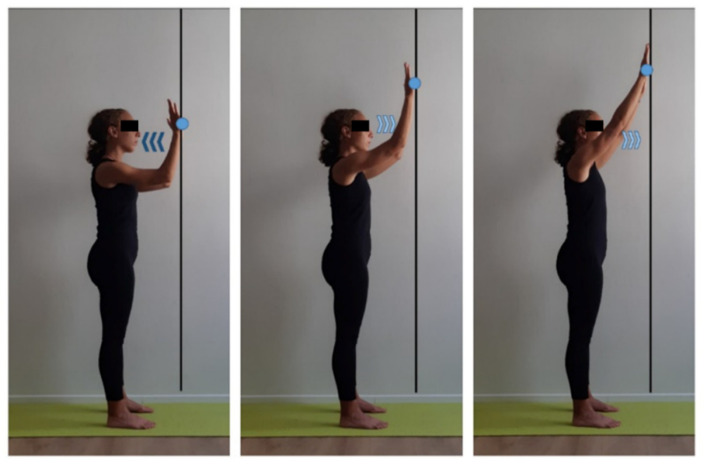
Scapulohumeral mobilization exercise in orthostatic position. Orthostatic position a step apart from the wall, lift both upper limbs and flex forearms placing the back of hands at gaze height (light blue dot), inhale (dark blue arrows). Exhale (light blue arrows) while performing an upward upper limbs extension. Focus on keeping trunk position, avoiding leaning back or forward. Three sets of 8 repetitions, 30 s recovery.

Patient had been educated to pair the exercise stretching phase with exhalation to reduce muscle tension by facilitating the release of muscular chains. Tactile and visual cues were provided in order to prevent compensative movements and to increase body perception/awareness. Once patient motor skills and correct execution of exercises were verified, APA_2_ was proposed. During this cycle (from 17th to 32nd session), on the basis that the patient had a proper postural control, the adapted exercise specialist added further goals to the protocol, including global body control, muscle strength improvement as well as trunk and lower limb stabilization exercises (Figure 8, Figure 9 and Figure 10).

**Figure 11:**
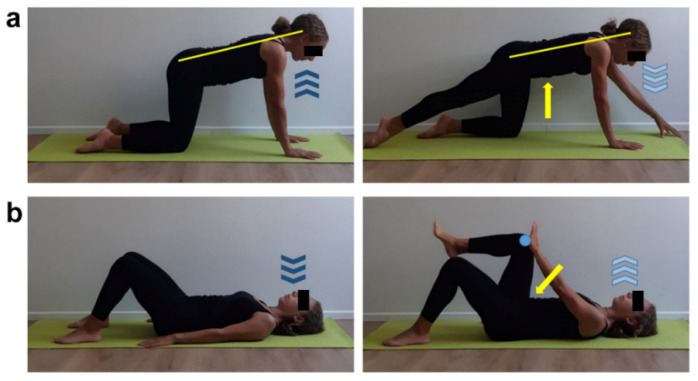
Thoraco-abdominal stabilization exercises. (**a**) Quadruped position, neutral spine attitude (yellow line), set hands shoulder-width apart and knees and feet hip-width apart. Inhale (dark blue arrows) and activate core muscles, then exhale (light blue arrows) while performing an extension of right lower limb and left upper limb keeping toes and fingertips in touch with the floor. Focus on hips-spine-shoulders-head alignment (yellow line), core muscles activation (yellow arrow) and feel upper limb stretching forward and lower limb backward. Two sets of 6 repetitions, 1 min recovery. (**b**) Supine position, rest straight arms by body sides and hand palms on the floor, flex both legs and place feet hip-width apart on the floor, inhale (dark blue arrows). Lift right lower limb keeping 90 degrees flexed leg and forming a 90 degrees angle between thigh and upper body, place left hand palm on right knee without touching it by fingers (light blue dot), exhale (light blue arrows) while performing an opposite push between hand palm and knee. Slowly get back to start position. Repeat with opposite hand/knee. Focus on performing opposite push until the exhalation phase is completed and perceiving an abdomen downward retraction (yellow arrow).

Even if the primary aim was an improvement of upper limb mobility, in order to strength the whole body muscle tone and coordination, bodyweight exercises (Figure 11) were alternated to exercises performed using small tools (e.g., sticks, sponge balls, low-resistance rubber bands), and circuit training mode was applied to sessions.

At the end of the APA pathway, the adapted exercise specialist proposed a cycle of AF aimed to achieve a further improvement of motor and functional skills by increasing workout’s intensity and loads. This part of the protocol allowed the patient to resume the sport activity she used to practice before oncological surgery sequelae occurred and to move from a healthcare environment to a recreational/sports one.

## 3. Results

Data concerning the functional test battery evaluation and self-reported questionnaires (NRS and SF-12) at the baseline, during (i.e., two cycles of eight-week APA) and after ending the structured PA protocol (i.e., eight-week AF) are shown in Table 1.

In particular, ROM values of non-surgical upper limb at baseline fell within the normal reference range and were preserved after the structured PA intervention (Table 1). Conversely, the positive effects on surgical upper limb’s ROM obtained through the first cycle of eight-week APA were further improved after the second one and post-AF. At the end of the whole PA protocol, ROM achieved normal values (Table 1). Similarly, muscle length test revealed that surgical shoulder mobility, which was heavily compromised at baseline, was improved after structured APA reaching the normal value of contralateral limb (Table 1). In addition, a further improvement in shoulder–arm mobility and posture, assessed by the PMm length test, was observed at the ending of the PA protocol (Table 1). Overall, these results demonstrated that after the structured PA intervention, a functional symmetry between surgical and non-surgical upper limb was achieved compared to baseline (Table 1). Moreover, at the baseline low back flexibility, assessed by sit and reach test, showed normal values that were preserved after the structured PA intervention (Table 1). We also observed a trend toward a decrease in low back pain after PA protocol, as well as a disappearance of surgical/non-surgical shoulder and cervical/dorsal pain (Table 1). Finally, as far as QoL assessment is concerned, both physical and mental components showed an improvement during and at the end of the PA protocol compared to the baseline (Table 1).

**Figure 12:**
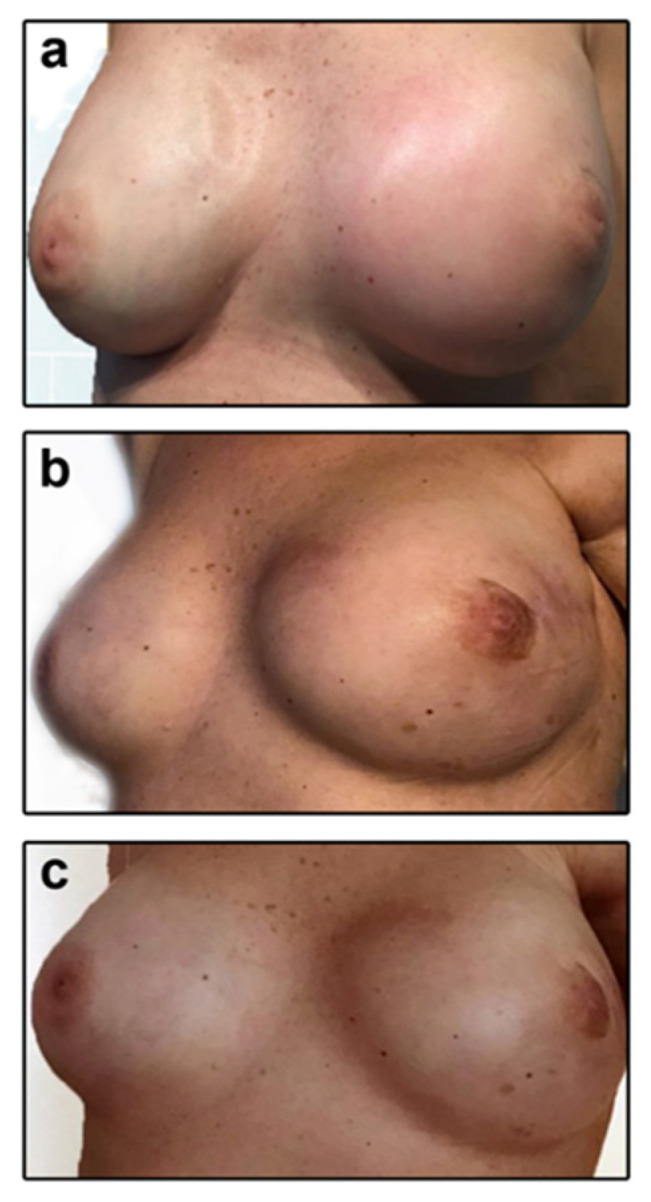
Seroma-related left breast swelling in a 56-year-old breast cancer survivor. (**a**) A diffuse edema with erythematous skin localized on the left side of breast was evident at baseline. (**b**) After 16-week adapted physical activity intervention, edema was reduced. (**c**) At the end of the whole physical activity protocol, the patient had no symptoms and signs of seroma. As shown in the picture, left breast looked like a normal one without breast swelling and skin changes similarly to the contralateral side.

As displayed in Figure 12, a progressive reduction in seroma-related left side breast swelling was observed during and at the end of the PA intervention compared to baseline.

## 4. Discussion

To our knowledge, this case report is the first directly presenting the effectiveness of a structured, well-planned, and adapted PA protocol to improve upper extremity functionality and QoL in a case of survivorship with seroma resulting from BC treatments.

Cancer survival improvements during the past few decades have resulted in a large and growing population of long-term BC survivors [2,15]. Hence, it is of primary importance to improve the health-related QoL of this population [3,5,6,11,15,16,17]. Of note, significant concerns exist regarding chronic upper extremity dysfunction as a long-term complication that can occur after BC treatments [3,4,5,13]. Upper limb functionality is essential in maintaining independent living and performing daily living activities. In addition, physical, mental, and social burdens may increase in case of upper extremity functional decrease, heavily jeopardizing QoL [3,4,5,13]. In particular, patients who underwent mastectomy frequently experience reduced shoulder ROM, pain syndromes and higher pectoralis muscle’s tightness than those who underwent breast-conserving surgery. Moreover, postoperative radiotherapy can complicate clinical signs and symptoms [3,4,5,6]. Seroma is also considered a frequent side effect of surgery [7,8,9,18].

In this context, the main goals of our APA protocol were to reduce breast seroma, and consequently eliminate pain and improve surgical shoulder mobility. Here, we reported a detailed description of the proposed exercises and methodology of our protocol which was organized in three cycles of eight-week training each, namely two cycles of APA (i.e., APA_1_ and APA_2_) followed by one of AF. The AF pathway was conceived with the intention of increasing the physical fitness achieved by taking part in the previous structured APA cycles, and then possibly starting or resuming a sport activity. In addition, AF was performed outside the Ce.Ri.On rehabilitation center, thus encouraging the subject to detach from a healthcare perspective and return to a normal life. It is remarkable that the structure of our PA protocol ensures tailored exercise prescription, constant supervision carried out by a qualified APA specialist, training progress evaluation toward target goals, and injury prevention. For this reason, each exercise was accurately described through biomechanical simple concepts and visually shown by the exercise specialist, and additional inputs by tactile and verbal cues were provided in order to improve the subject’s body perception/awareness. According to our previous studies [3,5,13,19], this methodology strengthens motivation and participation in the PA pathway. In fact, our subject attended all training sessions. In addition, it is remarkable that the baseline physical evaluation allowed the APA specialist to clearly identify the patient’s specific needs. Indeed, during assessment, a PMm retraction emerged as possible cause of seroma persistence. At the ending of the PA protocol, PMm length test showed better muscular/joint values and a consequent improvement in shoulder–arm mobility and postural control. Furthermore, our results confirm that a structured PA intervention may be effective not only in improving upper limb’s ROM and shoulder mobility, but also in reducing the marked inter-limb mobility asymmetry detected at baseline. Notably, our APA protocol was also effective in eradicating surgical shoulder pain. Pain is the most frequent (20–65%) adverse effect after BC treatment, negatively impacting QoL [15,20,21]. Increasing evidence suggests that pain syndromes following mastectomy are associated with myofascial dysfunction and nerve damage [15,20,21,22]. Therefore, attempts to reduce pain intensity are clinically relevant [22]. While we observed a complete resolution of cervical and dorsal pain, only a trend toward low back pain decrease was detected after our PA protocol. This can be related to the well-known evidence that non-specific low back pain is the most commonly reported musculoskeletal pain in adult females with a peak in the sixth decade [23]. Noteworthy, we herein show that the proposed PA pathway may significantly reduce seroma with an overall amelioration of QoL. Regarding seroma, physical examination after ending the PA protocol revealed a reduction in breast swelling with a similar aspect when compared to the contralateral side. Finally, concerning the QoL assessment, SF-12 physical and mental scores both increased after the PA pathway.

The multidimensional needs of BC survivors emphasize the necessity of a multidisciplinary perspective. Since the positive effects of structured and regular PA on psychophysical outcomes of BC survivors are widely demonstrated, qualified APA specialists should be included in the multidisciplinary team in order to guarantee a comprehensive approach to oncological patient care.

## 5. Conclusions

A structured and planned PA pathway, tailored to the individual patient’s needs and supervised by a qualified APA specialist, may represent an effective strategy to improve upper limb functionality and QoL in cases of seroma as severe sequela of BC treatments. Our encouraging findings need to be further explored and confirmed applying the protocol to a larger sample. Notably, this case report also highlights the urgent need of developing specific APA guidelines aimed at alleviating seroma-related side effects in BC survivors.

## Figures and Tables

**Table 1:** Fitness tests, pain intensity and quality of life scores at baseline and after the end of either two adapted physical activity (APA_1_ and APA_2_) or adapted fitness (AF) protocols.

Variables	Baseline	Post-APA_1_	Post-APA_2_	Post-AF
Surgical limb ROM				
Flexion (°)	145	155	180	180
Extension (°)	45	45	45	45
External rotation (°)	55	60	75	90
Abduction (°)	110	120	180	180
Non-surgical limb ROM				
Flexion (°)	180	180	180	180
Extension (°)	45	45	45	45
External rotation (°)	80	90	90	90
Abduction (°)	180	180	180	180
Surgical shoulder mobility (cm)	29	15	0	0
Non-surgical shoulder mobility (cm)	0	0	0	0
Surgical shoulder PMm length test (cm)	13	10.5	9	8
Non-surgical shoulder PMm length test (cm)	8	8	8	8
Sit and reach (cm)	0	0	0	0
Perception of pain (NRS)				
Surgical shoulder pain	8	6	0	0
Non-surgical shoulder pain	5	3	0	0
Cervical pain	2	0	0	0
Dorsal pain	8	6	4	0
Lumbar pain	10	8	4	4
Quality of life (SF-12)				
Physical	45.92	47.79	51.59	51.70
Mental	59.37	60.57	59.67	59.53

Abbreviations: ROM, range of motion; PMm, pectoralis minor muscle; NRS, Numerical rating scale; SF-12, Short Form-12.
